# Clinical Outcomes After Same-Agent Double-Dose Anti-VEGF Escalation in Eyes with a Suboptimal Response to Standard-Dose Treatment for Exudative Macular Diseases: A Real-World Pre–Post Study

**DOI:** 10.3390/jcm15155898

**Published:** 2026-07-28

**Authors:** Qiumei Gu, Hongyi Liu, Yifan Xie, Fang Lu, Ziyan He, Yilong Chen, Jiacen Wan, Xiaoshuang Jiang

**Affiliations:** 1 Department of Ophthalmology, West China Hospital, Sichuan University, Chengdu 610041, China; 2 Department of Eye and Vision Sciences, University of Liverpool, Liverpool L7 8TX, UK; 3 Department of Computer Science, Faculty of Environment, Science and Economy, University of Exeter, Exeter EX4 4RN, UK; 4 West China Biomedical Big Data Center, West China Hospital, Sichuan University, Chengdu 610041, China; 5 Med-X Center for Informatics, Sichuan University, Chengdu 610041, China; 6 Xizang Hospital of West China Hospital, Sichuan University, Lhasa 850000, China

**Keywords:** anti-VEGF, exudative macular diseases, dose escalation, treatment intensification, real-world study

## Abstract

**Background/Objectives:** A substantial proportion of eyes with exudative macular diseases show a suboptimal response to standard-dose anti-vascular endothelial growth factor (anti-VEGF) therapy, and evidence regarding clinical outcomes after same-agent double-dose escalation remains limited. We evaluated the functional and anatomical outcomes of same-agent double-dose anti-VEGF therapy in such eyes. **Methods:** In this observational real-world cohort study, we retrospectively reviewed eyes with exudative macular diseases that were switched to same-agent double-dose anti-VEGF therapy because of a suboptimal response to prior standard-dose treatment. Eligibility required clinician-assessed persistent or recurrent exudative activity during the standard-dose treatment course despite at least three prior anti-VEGF injections, based on the longitudinal treatment and imaging history. Insufficient visual response and inadequate durability were considered additional, potentially overlapping features supporting dose escalation. Primary outcomes were functional and anatomical changes after switching. Secondary outcomes included injection interval, time-to-event outcomes, and associated baseline factors. **Results:** A total of 104 patients (111 eyes) were included. Mixed-effects modeling showed a small longitudinal improvement in BCVA of 0.007 logMAR per month (*p* = 0.028) and an estimated CST decrease of approximately 2.1% per month (*p* < 0.001). In visit-specific analyses, BCVA did not differ significantly from baseline at the first three post-switch visits and differed significantly only at the final follow-up (*p* = 0.004); however, the median BCVA was 0.70 logMAR at both baseline and final follow-up, and the median within-eye change was 0.00 logMAR (IQR, −0.25 to 0.10). CST was significantly lower than baseline at all post-switch visits (all *p* < 0.01). Among the 73 eyes with calculable paired interval data, injection intervals did not differ significantly between the standard-dose and double-dose phases (*p* = 0.289). Baseline pigment epithelial detachment was associated with a less favorable BCVA change (*p* = 0.002), and worse baseline visual acuity was associated with a higher risk of subretinal fibrosis (hazard ratio, 2.854; *p* = 0.002). **Conclusions:** In this uncontrolled pre–post cohort, anatomical changes after same-agent double-dose escalation were more consistent than functional changes. The BCVA change was statistically detectable but small in magnitude and of uncertain clinical relevance. No significant interval extension was observed among the 73 eyes with complete paired interval data during the relatively short follow-up.

## 1. Introduction

Exudative macular diseases, including neovascular age-related macular degeneration (nAMD), diabetic macular edema (DME), retinal vein occlusion-related cystoid macular edema (RVO-CME), and other forms of choroidal neovascularization (CNV), are major causes of central vision loss [1,2,3]. Intravitreal anti-vascular endothelial growth factor (anti-VEGF) therapy has become the standard treatment for many of these conditions and can provide substantial functional and anatomical benefits [4,5]. However, despite its established efficacy, a considerable proportion of patients show a suboptimal response to standard-dose therapy in routine clinical practice, as reflected primarily by persistent or recurrent exudative activity despite treatment, sometimes accompanied by limited visual improvement or inadequate durability [6,7]. These patients represent an important therapeutic challenge, as continued standard-dose treatment may provide only limited incremental benefit. Same-agent dose escalation has therefore been explored as a potential treatment-intensification strategy.

The rationale for high-dose anti-VEGF therapy is that increased drug exposure may enhance VEGF suppression, thereby improving anatomical drying and potentially facilitating functional recovery in eyes that respond inadequately to conventional dosing [8,9,10]. However, real-world evidence regarding clinical outcomes after this approach remains limited, particularly in heterogeneous cohorts of exudative macular diseases encountered in routine clinical practice [11,12,13]. In our clinical setting, the double-dose regimens evaluated in this study were operationally feasible within a single vial or syringe. This practical feasibility may be particularly relevant in settings where approved high-dose or dual-pathway therapies are unavailable. Accordingly, same-agent dose escalation should be considered within a broader treatment framework that includes approved high-dose formulations and dual-pathway agents [14,15]. Its potential role may be limited to selected eyes with a partial response to the original agent, with treatment selection guided by the presumed mechanism of suboptimal response, drug availability, and patient-specific clinical considerations. In addition to characterizing visual and anatomical outcomes, it is clinically important to determine whether injection intervals change after dose escalation and whether baseline factors are associated with subsequent outcomes.

We therefore conducted an observational real-world study based on retrospective clinical data to evaluate the outcomes of switching from standard-dose to same-agent double-dose anti-VEGF therapy in eyes with exudative macular diseases that had shown a suboptimal response to prior treatment. Specifically, we assessed changes in best-corrected visual acuity (BCVA), central subfield thickness (CST), and OCT biomarkers after switching, compared injection intervals between the standard-dose and double-dose phases, and explored baseline factors associated with post-switch changes and time-to-event outcomes.

## 2. Materials and Methods

### 2.1. Study Design and Data Source

This was an observational, single-center, real-world cohort study based on retrospective review of routinely collected clinical data from patients who received intravitreal anti-vascular endothelial growth factor (anti-VEGF) therapy at the Department of Ophthalmology, West China Hospital, Sichuan University. The study was approved by the Ethics Committee of West China Hospital, Sichuan University (approval no. 2025-682) and was conducted in accordance with the Declaration of Helsinki. All patients provided written informed consent for each intravitreal injection, and same-agent double-dose treatment was administered in routine clinical practice at the discretion of the treating retina specialist after discussion with the patient, with specific informed consent for the off-label double-dose treatment obtained from each patient. The approved study protocol covered the retrospective review of eyes that had received standard-dose and same-agent double-dose anti-VEGF therapy in routine clinical practice, and all analyzed data were anonymized and de-identified. The study was registered in the Chinese Clinical Trial Registry (registration no. ChiCTR2500102705). No formal sample size calculation was performed; all eligible eyes during the study period were included in the analysis.

We retrospectively reviewed the medical records and imaging data of patients seen between July 2020 and January 2025, including best-corrected visual acuity (BCVA), intraocular pressure (IOP), and optical coherence tomography (OCT) findings. At our institution, standard-dose anti-VEGF therapy was generally administered using a treat-and-extend strategy, typically beginning with three consecutive monthly intravitreal injections followed by extension or shortening of the treatment interval according to disease activity. The anti-VEGF agents used included ranibizumab (0.5 mg; Novartis Pharma Stein AG, Stein, Switzerland), conbercept (0.5 mg; Chengdu Kanghong Biotechnologies Co., Ltd., Chengdu, China), and aflibercept (2 mg; Bayer AG, Berlin, Germany). Eyes that were judged to have a suboptimal response to standard-dose treatment were subsequently switched to double-dose therapy with the same agent, namely ranibizumab 1.0 mg, conbercept 1.0 mg, and aflibercept 4 mg. No protocol-mandated loading phase was restarted after dose escalation. The first double-dose injection was administered at the time of switching, and subsequent treatment intervals continued to be adjusted according to disease activity within the existing treat-and-extend strategy.

**Figure 1:**
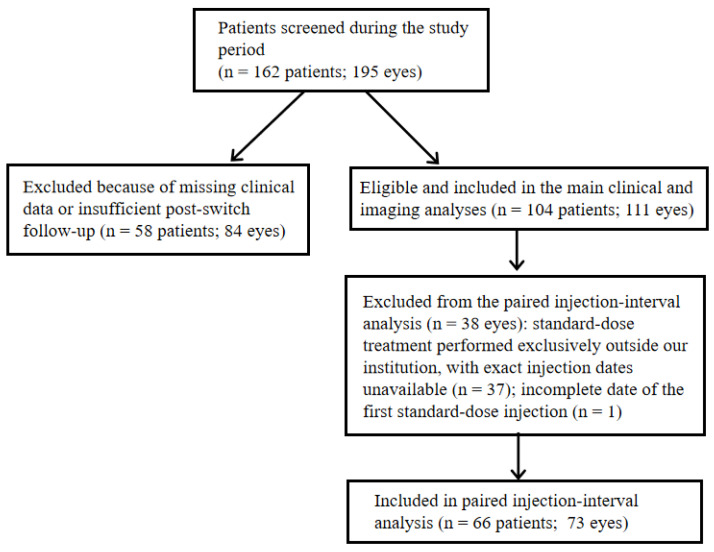
Study flowchart.

Eyes were eligible if they met all of the following criteria: (1) receipt of at least three prior standard-dose intravitreal anti-VEGF injections before switching, as verified at the pre-switch consultation using the patient-reported treatment history documented in the medical record, available outside treatment records, and injection-related documentation retained by the patient; (2) clinician-assessed persistent or recurrent exudative activity during the standard-dose treatment course despite these injections, based on the longitudinal treatment and imaging history; and (3) switching to double-dose anti-VEGF therapy with completion of at least four follow-up visits. Exudative activity included persistent or recurrent intraretinal fluid, subretinal fluid, macular edema, hemorrhage, or other clinically meaningful exudative signs judged by the treating physician. The assessment was based on the longitudinal standard-dose treatment course and did not require active fluid to be present at the single pre-switch OCT assessment if recurrent activity or inadequate durability had been documented during the preceding treatment course. Insufficient visual improvement and inadequate durability were considered additional, potentially overlapping features supporting dose escalation, but were not independent alternatives to anatomical activity. The minimum exposure threshold of three prior standard-dose injections was verified for all included eyes. This minimum threshold was selected to ensure completion of at least an initial anti-VEGF treatment course before dose escalation while retaining the pragmatic real-world nature of the cohort; the term ‘treatment-resistant’ was not used as an eligibility designation. However, for eyes previously treated outside our institution, the exact cumulative number and dates of externally administered injections were not consistently recoverable. Uncertain external injection counts were therefore not imputed or summarized quantitatively. Eyes were excluded if they met any of the following criteria: (1) missing clinical data or insufficient post-switch follow-up data, precluding completion of the prespecified outcome assessments; and (2) coexisting ocular diseases that could substantially affect the evaluated outcomes, such as severe glaucoma, retinal detachment, or macular hole. Post-switch ocular adverse events were assessed as safety outcomes and were not used as exclusion criteria. Outcome-specific analyses were restricted to eyes with the data required for the corresponding prespecified analysis. The study flow is shown in Figure 1.

### 2.2. Data Collection

Baseline demographic and clinical data, including age, sex, BCVA, and IOP, were collected retrospectively from the medical records. BCVA was converted to the logarithm of the minimum angle of resolution (logMAR) for statistical analysis. All included eyes underwent routine ophthalmic examinations and optical coherence tomography (OCT) using a Cirrus 5000 device (Carl Zeiss Meditec, Inc., Dublin, CA, USA). OCT was used to evaluate central subfield thickness (CST) and related anatomical features, including intraretinal fluid (IRF), subretinal fluid (SRF), pigment epithelial detachment (PED), hyperreflective foci (HF), macular atrophy (MA), and subretinal fibrosis. These clinical and imaging data, including BCVA, IOP, CST, and OCT morphological features, were obtained at the pre-switch baseline and at each post-switch follow-up visit from the hospital medical record and imaging archive systems. Medical records were also reviewed for documented ocular adverse events after switching to double-dose therapy, including intraocular inflammation, retinal vasculitis, occlusive retinal vasculitis, endophthalmitis, clinically relevant IOP elevation, and treatment discontinuation attributable to these events.

### 2.3. Statistical Analysis

All statistical analyses were performed using R software (version 4.1.1; R Foundation for Statistical Computing, Vienna, Austria). Continuous variables are presented as median (interquartile range [IQR]) or mean ± standard deviation (SD), as appropriate, and categorical variables are presented as counts and percentages. For visit-specific paired comparisons between the pre-switch baseline and each post-switch follow-up visit, including comparisons of BCVA, CST, and IOP, the Wilcoxon signed-rank test was used for continuous variables and the McNemar test for categorical variables. Injection intervals were calculated separately for each eye during the standard-dose and double-dose treatment phases. For the standard-dose phase, the mean injection interval was calculated as the number of days from the first standard-dose injection documented at our institution to the first double-dose injection, divided by the number of consecutive injection intervals during this period. This number of intervals equaled the number of standard-dose injections administered at our institution before switching, because the interval between the final standard-dose injection and the first double-dose injection was included. For the double-dose phase, the mean injection interval was calculated across four consecutive intervals defined by five injection dates: the switching injection and the first, second, third, and final post-switch injection visits. The paired injection-interval analysis was conducted as a complete-case analysis and included only eyes with calculable intervals during both treatment phases. Inclusion required sufficiently complete injection-date records rather than successful interval extension during the standard-dose phase. Potential selection bias was assessed by comparing available characteristics between eyes included in and excluded from the paired analysis. Continuous variables were summarized as medians and interquartile ranges, and between-group imbalance was quantified using absolute Cliff’s delta. Categorical variables were summarized as counts and percentages, and imbalance was quantified using the absolute standardized mean difference (SMD). Cliff’s delta was interpreted as negligible (<0.147), small (0.147 to <0.330), medium (0.330 to <0.474), or large (≥0.474), whereas an absolute SMD of ≥0.20 was considered potentially meaningful.

To characterize longitudinal changes after same-agent double-dose escalation, mixed-effects models were fitted. Time since dose escalation, measured in months, was included as a continuous fixed effect. Eye-specific random intercepts and random slopes for time were included to account for repeated measurements within eyes. A linear mixed-effects model for BCVA (logMAR) was fitted using the lmer() function in the lme4 package. Because CST showed a right-skewed distribution with heteroscedasticity, a gamma mixed-effects model with a log link was fitted using the glmmTMB() function. No additional fixed effects were included.

Time-to-event outcomes were analyzed using Kaplan–Meier methods and Cox proportional hazards regression models. For the complete-OCT-dryness analysis, the event was defined as the first post-switch OCT assessment demonstrating the absence of intraretinal fluid (IRF), subretinal fluid (SRF), and pigment epithelial detachment (PED). Time zero was the date of the first double-dose injection, and eyes without the event were censored at their last available OCT assessment. Other time-to-event outcomes were time to the first observed macular atrophy and subretinal fibrosis. A descriptive analysis of complete OCT dryness by disease category was performed among eyes with at least one of IRF, SRF, or PED at the pre-switch baseline. Given the small subgroup sizes, no formal between-disease hypothesis testing was performed. Multivariable linear regression analyses were performed to examine baseline factors associated with changes in BCVA (logMAR) and CST from baseline to the final follow-up. The exploratory multivariable linear regression models included clinically relevant baseline covariates, including age, sex, baseline BCVA or CST, intraocular pressure (IOP), subretinal fluid (SRF), pigment epithelial detachment (PED), MA, and subretinal fibrosis. All statistical tests were two-sided, and a *p* value < 0.05 was considered statistically significant.

## 3. Results

### 3.1. Baseline Characteristics

The baseline demographic and clinical characteristics are summarized in Table 1. A total of 104 patients (111 eyes) were included. All 111 eyes had clinician-documented persistent or recurrent exudative activity during the preceding standard-dose treatment course. The median age was 71.0 years (IQR, 60.5–78.0), and 69 eyes (62.2%) were from male patients. The cohort mainly comprised eyes with neovascular age-related macular degeneration (nAMD; 87/111, 78.4%), followed by diabetic macular edema (DME; 10/111, 9.0%), retinal vein occlusion-related cystoid macular edema (RVO-CME; 8/111, 7.2%), and other CNV (6/111, 5.4%). The initial same-agent double-dose regimens were aflibercept in 72 eyes (64.9%), conbercept in 28 eyes (25.2%), and ranibizumab in 11 eyes (9.9%) (Table 1). Aflibercept-treated nAMD was the largest diagnosis–agent subgroup, accounting for 61 of 111 eyes (55.0%). Right eyes accounted for 56.8% of the cohort. At baseline, the median best-corrected visual acuity (BCVA) was 0.70 logMAR (IQR, 0.40–1.00), and the median intraocular pressure (IOP) was 14.0 mmHg (IQR, 12.20–15.60). Before dose escalation, the median documented duration of standard-dose treatment was 8 months (IQR, 2–16). The median post-switch follow-up duration was 6.9 months (IQR, 5.7–9.3).

### 3.2. Visit-Specific Functional and Anatomical Outcomes After Same-Agent Double-Dose Escalation

**Figure 2:**
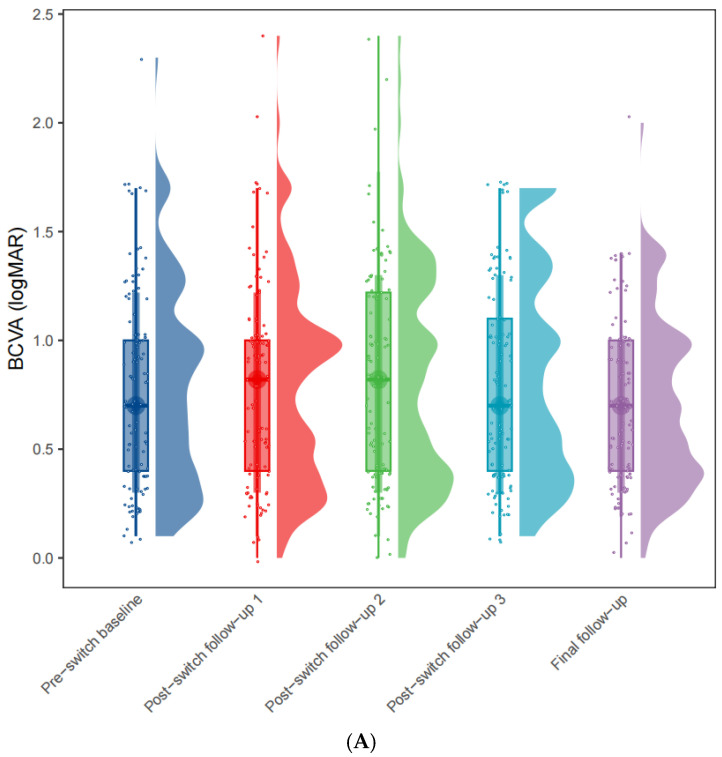

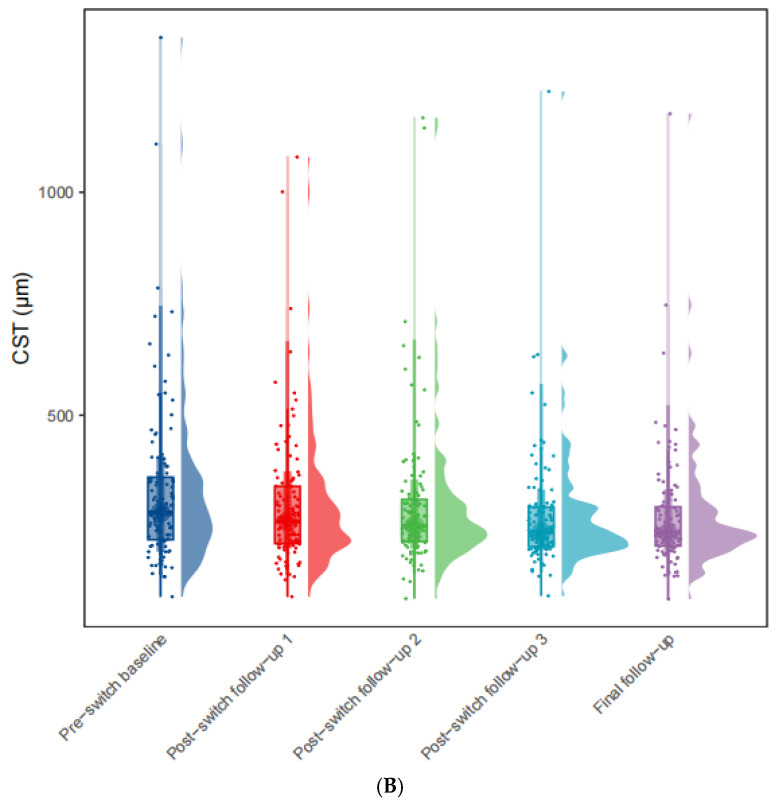
Distributions of BCVA and CST at the pre-switch baseline and post-switch follow-up visits after same-agent double-dose escalation: (**A**) BCVA (logMAR); (**B**) CST.

The visit-specific BCVA trajectory was non-monotonic. Compared with the pre-switch baseline, BCVA did not differ significantly at the first, second, or third post-switch follow-up visits (*p* = 0.776, 0.608, and 0.685, respectively) and differed significantly only at the final follow-up (*p* = 0.004). However, the median BCVA was 0.70 logMAR at both baseline and final follow-up, and the median within-eye change was 0.00 logMAR (IQR, −0.25 to 0.10) (Table 2; Figure 2A).

Median central subfield thickness (CST) was lower than the pre-switch baseline at all post-switch visits. The median CST decreased from 282 μm (IQR, 221–361) at baseline to 262 μm (IQR, 212.4–340.5) at the first follow-up, 252 μm (IQR, 216.85–311.00) at the second follow-up, 238 μm (IQR, 205.5–294.0) at the third follow-up, and 236 μm (IQR, 207.0–294.5) at the final follow-up. Compared with baseline, the reductions at all follow-up visits were statistically significant (*p* = 0.002, 0.004, *p* < 0.001, and *p* < 0.001, respectively). At the final follow-up, the median change in CST from baseline was −19 μm (IQR, −98.0 to 3.5) (Table 2; Figure 2B).

The proportion of eyes with IRF decreased from 55.9% at baseline to 48.6% at the first follow-up and 45.0% at the final follow-up, with significant differences at the first and final visits (*p* = 0.013 and *p* = 0.006, respectively). The proportion with SRF decreased from 44.1% at baseline to 34.2% at the first follow-up (*p* = 0.046), whereas the differences at the second, third, and final visits were not statistically significant (*p* = 0.054, 0.188, and 0.059, respectively). The proportion with PED decreased from 28.8% at baseline to 22.5% at the first follow-up (*p* = 0.023), with no significant differences at subsequent visits (Table 2). Mean ± standard deviation values for the functional and anatomical outcomes at each time point are provided in Supplementary Table S1. In exploratory analyses stratified by the initial escalation agent, neither BCVA change nor CST change from baseline to final follow-up differed significantly among the three agents (*p* = 0.218 and *p* = 0.134, respectively; Supplementary Table S8).

### 3.3. Injection Interval Comparison

Of the 111 included eyes, 73 had calculable injection intervals during both treatment phases and were included in the paired complete-case analysis. Thirty-eight eyes were excluded because exact injection dates for standard-dose treatment performed exclusively outside our institution were unavailable (n = 37), or because the date of the first standard-dose injection was incomplete (n = 1). Effect-size comparisons indicated imbalances in several available characteristics. Aflibercept was used as the initial escalation agent in 56.2% of included eyes and 81.6% of excluded eyes (absolute SMD, 0.57), whereas conbercept was used in 31.5% and 13.2%, respectively (absolute SMD, 0.45). The median documented standard-dose treatment duration was 5.9 months in the included group and 12.2 months in the excluded group (absolute Cliff’s δ, 0.40) (Supplementary Table S9). Among the 73 included eyes, the median injection interval did not differ significantly between the standard-dose and double-dose phases (55 days [IQR, 38–85] vs. 52 days [IQR, 43–69]; *p* = 0.289) (Supplementary Table S2).

### 3.4. Longitudinal Mixed-Effects Analyses

To characterize longitudinal changes after same-agent double-dose escalation, mixed-effects models were fitted (Supplementary Tables S3 and S4). The linear mixed-effects model estimated a small decrease in logMAR BCVA of 0.007 per month (SE, 0.003; *p* = 0.028) (Supplementary Table S3). The gamma mixed-effects model showed that CST decreased significantly over time (β = −0.021297; exp[β] = 0.979; SE, 0.005112; *p* < 0.001), corresponding to an estimated monthly reduction of approximately 2.1% (Supplementary Table S4). The mixed-effects models indicated statistically significant longitudinal changes in both BCVA and CST. However, the earlier visit-specific detectability of the CST change was derived from the comparisons in Table 2: CST was significantly lower from the first post-switch follow-up, whereas BCVA differed significantly from baseline only at the final follow-up.

### 3.5. Time-to-Event Analyses

**Figure 3:**
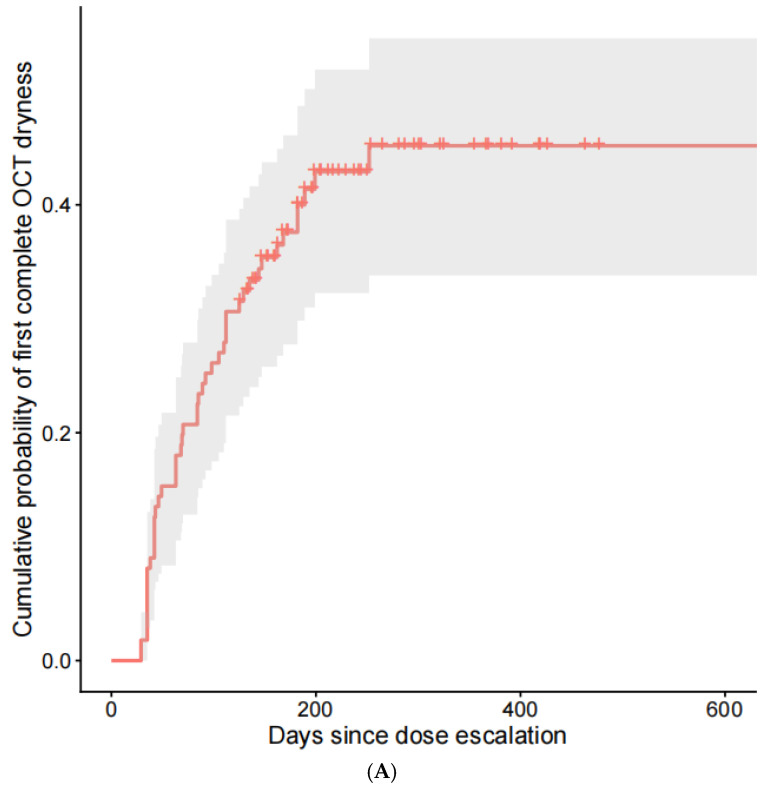

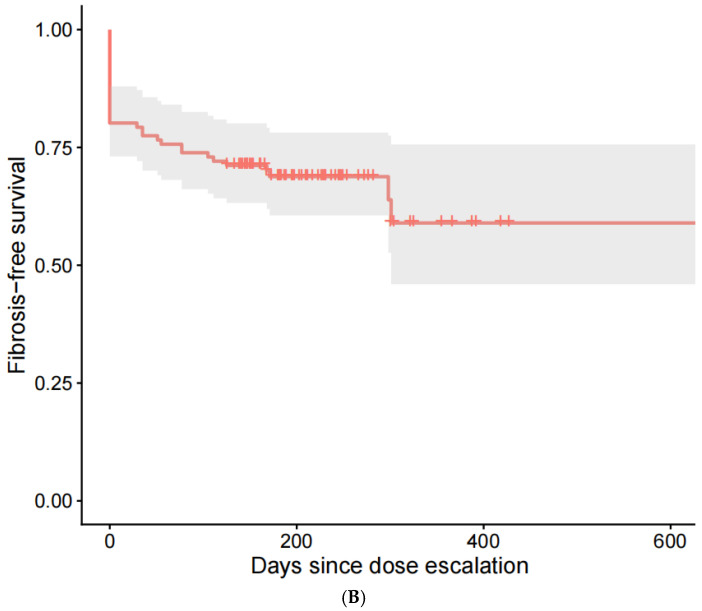
Kaplan–Meier analyses after same-agent double-dose escalation. (**A**) Cumulative probability of first complete OCT dryness, defined as the absence of intraretinal fluid, subretinal fluid, and pigment epithelial detachment. (**B**) Fibrosis-free survival. Shaded areas represent 95% confidence intervals, and tick marks indicate censored observations.

Kaplan–Meier analysis showed that the cumulative probability of first complete OCT dryness increased mainly during the early to middle follow-up period and approached a plateau after approximately 200–300 days (Figure 3A). MA events were infrequent during follow-up (Supplementary Figure S1), whereas subretinal fibrosis occurred more commonly, with approximately 60% of eyes remaining free of subretinal fibrosis at later follow-up (Figure 3B). Cox proportional hazards regression analysis showed that no baseline factors were significantly associated with first complete OCT dryness or time to first observed MA (Supplementary Table S5, Panels A and B). In contrast, worse baseline visual acuity was independently associated with a higher risk of subretinal fibrosis (HR 2.854, 95% CI 1.493–5.453; *p* = 0.002) (Supplementary Table S5, Panel C). Among the 95 eyes with at least one of IRF, SRF, or PED at the pre-switch baseline, 32 eyes (33.7%) achieved complete OCT dryness during follow-up. Complete OCT dryness was observed in 25 of 73 eyes with nAMD (34.2%), 2 of 8 eyes with DME (25.0%), 4 of 8 eyes with RVO-CME (50.0%), and 1 of 6 eyes with other CNV (16.7%) (Supplementary Table S7). No formal between-disease comparisons were performed because of the small subgroup sizes.

### 3.6. Multivariable Linear Regression Analyses

In the multivariable linear regression model for BCVA change from the pre-switch baseline to the final follow-up, baseline pigment epithelial detachment (PED) was associated with a less favorable BCVA change (β = 0.198; 95% CI, 0.073–0.323; *p* = 0.002), whereas no other included baseline variable was significantly associated with BCVA change (Supplementary Table S6, Panel A). The model had an R^2^ of 0.12 and an adjusted R^2^ of 0.05.

In the multivariable linear regression model for CST change from the pre-switch baseline to the final follow-up, none of the included baseline variables was significantly associated with CST change (Supplementary Table S6, Panel B). The model had an R^2^ of 0.07 and an adjusted R^2^ of −0.001.

### 3.7. Safety Outcomes

Median IOP was 14.0 mmHg (IQR, 12.20–15.60) at the pre-switch baseline and 13.9 mmHg (IQR, 12.25–15.20), 14.2 mmHg (IQR, 12.40–16.05), 14.2 mmHg (IQR, 12.75–15.30), and 14.1 mmHg (IQR, 12.35–15.75) at the first, second, third, and final post-switch follow-up visits, respectively. IOP did not differ significantly from baseline at any post-switch visit (*p* = 0.700, 0.126, 0.152, and 0.151, respectively). No eye discontinued same-agent double-dose anti-VEGF treatment because of a serious ocular adverse event. No cases of intraocular inflammation, retinal vasculitis, occlusive retinal vasculitis, or endophthalmitis were documented during follow-up.

## 4. Discussion

We conducted this study to characterize clinical outcomes after same-agent double-dose anti-VEGF escalation in eyes with a suboptimal response to standard-dose treatment. In the visit-specific analyses, CST was significantly lower than the pre-switch baseline at all post-switch visits, and IRF prevalence was lower at the first and final visits. In contrast, BCVA did not differ significantly from baseline at the first three post-switch visits and differed significantly only at the final follow-up; however, median BCVA remained 0.70 logMAR, with a median within-eye change of 0.00 logMAR. The mixed-effects models similarly estimated a small longitudinal BCVA change of 0.007 logMAR per month and a CST reduction of approximately 2.1% per month. Overall, the observed anatomical changes were more consistent than the functional changes.

These findings should be interpreted within the limitations of an uncontrolled within-eye pre–post design. Eyes were selected for dose escalation at a time of suboptimal disease control, creating extreme-value selection and a substantial risk of regression to the mean. Although the group median CST decreased from 282 μm at baseline to 236 μm at the final follow-up, the proportion of this difference attributable specifically to dose escalation cannot be estimated without a contemporaneous control group. All eyes also continued to receive anti-VEGF injections after switching; therefore, the observed changes may reflect a combination of continued treatment, natural fluctuations in exudative activity, heterogeneous visit timing, and regression to the mean. The results should consequently be interpreted as within-eye temporal associations after dose escalation rather than evidence of a dose-specific causal effect.

The more consistent anatomical than functional pattern observed in our cohort is concordant with directly comparable prior work. The DIANA study evaluated aflibercept dose escalation from 2 mg to 4 mg in nAMD and similarly reported greater CMT reduction and a lower incidence of IRF without a corresponding visual benefit [13]. Our findings are consistent with this previously reported anatomical–functional dissociation and provide complementary observations in a broader real-world cohort encompassing multiple exudative macular diseases and three same-agent dose-escalation regimens. Similar patterns have been reported in other intensified anti-VEGF studies: Broadhead et al. observed anatomical improvement without significant visual gain after higher-dose anti-VEGF treatment, Bala et al. reported reduced retinal fluid with largely stable visual acuity after aflibercept 8 mg, and Qian et al. found greater early PED reduction but only a transient visual difference after double-dose conbercept [11,12,16]. Collectively, these findings suggest that anatomical changes after intensified anti-VEGF exposure may be more readily detectable than clinically meaningful functional changes.

The limited additional visual change after dose escalation may partly reflect a ceiling effect from prior treatment rather than necessarily indicating a long disease course or advanced irreversible macular remodeling. In nAMD, a substantial component of the early functional response may occur during the initial three-injection loading phase of anti-VEGF therapy [17]. Eyes in the present study may therefore have already realized part of their functional potential during the preceding standard-dose phase, leaving limited room for further BCVA improvement despite additional anatomical drying. Functional recovery also depends on baseline visual reserve, retinal fluid characteristics, and macular structural integrity [18,19,20]. Chronic photoreceptor or retinal pigment epithelium damage may have limited recovery in some eyes; however, these features were not systematically quantified and should not be assumed to characterize the entire cohort. The non-monotonic visit-specific BCVA trajectory and the median final within-eye change of 0.00 logMAR further argue against interpreting the functional change as gradual or uniform.

Among the OCT-related anatomical biomarkers, the reduction in IRF prevalence was more consistent than the changes in SRF and PED. This pattern is broadly consistent with previous imaging studies demonstrating compartment-specific differences in anti-VEGF response. Simader et al. reported that intraretinal cysts resolved most rapidly, followed by SRF, whereas PED decreased more slowly [17]; previous evidence has also associated baseline IRF and PED with less favorable visual outcomes, while SRF may be associated with more favorable visual change in some settings [18,19,20]. In our cohort, IRF prevalence was significantly lower than baseline at the first and final follow-up visits, whereas SRF and PED showed less consistent visit-specific differences. These findings indicate that the observed morphological changes differed across lesion compartments rather than representing a uniform anatomical response. PED may reflect a more complex or chronic lesion configuration and may be less responsive to dose escalation alone. Consistent with this interpretation, baseline PED was independently associated with a less favorable BCVA change from baseline to the final follow-up. However, because the cohort included nAMD, DME, RVO-CME, and other CNV, heterogeneity in pathophysiology, agent exposure, and treatment response is expected. The findings should therefore be interpreted as descriptive real-world associations in a heterogeneous cohort rather than disease-specific efficacy estimates.

During the median post-switch follow-up of 6.9 months, no significant extension of the injection interval was observed among the 73 eyes with calculable paired interval data. This numerical pattern differs from that reported in the DIANA study, which found a longer median remission interval with aflibercept 4 mg than with 2 mg (5 vs. 3 months), although the difference was not statistically significant (*p* = 0.452) [13]. The findings are not directly comparable because DIANA evaluated remission intervals under a PRN regimen, whereas the present study compared eye-level mean inter-injection intervals within a treat-and-extend framework. Differences in disease composition, anti-VEGF agents, follow-up duration, and outcome definitions may also have contributed to the divergent numerical patterns.

The paired interval findings should also be interpreted in light of non-random data availability. The 73-eye complete-case subgroup differed from the 38 excluded eyes in several available demographic, clinical, and treatment characteristics. Notable imbalances included initial aflibercept use (absolute SMD, 0.57), initial conbercept use (absolute SMD, 0.45), and documented standard-dose treatment duration (absolute Cliff’s δ, 0.40) (Supplementary Table S9). One possible explanation is that eyes with sufficiently complete longitudinal records may have undergone more structured interval adjustment before escalation and may therefore have had less remaining scope for further extension, potentially biasing the analysis against detecting interval prolongation. However, because standard-dose interval data were unavailable for the excluded eyes and several characteristics were imbalanced, the net direction and magnitude of this bias cannot be determined with certainty. The absence of significant interval extension should therefore be regarded as a complete-case finding restricted to the selected 73-eye subgroup rather than evidence that dose escalation cannot improve durability.

The relatively short follow-up may also have been insufficient to capture later interval extension. In routine treat-and-extend practice, clinicians may initially prioritize suppression of exudative activity and anatomical stabilization before extending treatment intervals. Previous intensified anti-VEGF studies have similarly reported heterogeneous durability outcomes. Broadhead et al. found that eyes receiving higher-dose anti-VEGF therapy continued to require injections at intervals of approximately 5.7–6.4 weeks, whereas Bala et al. reported some interval extension with aflibercept 8 mg but noted that many eyes remained unable to maintain intervals of at least 8 weeks because of persistent macular fluid [11,16]. By contrast, Funatsu et al. observed reduced injection frequency in a subset of eyes with DME treated with aflibercept 8 mg under a PRN regimen [21]. These findings suggest that potential treatment-burden reduction may depend on the underlying disease, treatment regimen, follow-up duration, and degree of anatomical stabilization. Longer prospective follow-up using a predefined extension strategy is needed to determine whether intervals can subsequently be extended.

Same-agent double-dose escalation should be interpreted in relation to contemporary treatment alternatives. Aflibercept 8 mg is an approved high-concentration formulation supported by randomized evidence for extended dosing intervals [14], whereas faricimab provides dual inhibition of VEGF-A and angiopoietin-2 and represents a mechanistically distinct alternative [15]. Accordingly, further escalation of a VEGF-only agent may be less likely to address residual disease activity driven by non-VEGF pathways, including angiopoietin-2 or fibrotic mechanisms [15,22,23,24]. Switching to faricimab has also been associated with anatomical improvement in some previously treated or treatment-resistant eyes [25,26,27]. However, recent evidence suggests that further dose escalation may encounter a pharmacological ceiling, as increasing faricimab from 6 mg to 12 mg provided no significant additional anatomical benefit in a small group of nonresponding eyes [28].

In our clinical practice, same-agent double dosing was generally considered in selected eyes that retained a partial anatomical response to the original agent but had insufficient disease control; switching to another agent was considered when the response remained inadequate. During the study period, aflibercept 8 mg had not yet been approved in China, with approval for nAMD occurring after study completion in May 2025 [29]. Faricimab was not used in this cohort and was therefore not directly evaluated. Same-agent double-dose escalation should consequently be regarded as a context-dependent, off-label option when approved high-dose or dual-pathway alternatives are unavailable or clinically unsuitable. The regimens evaluated in this study were operationally feasible within a single vial or syringe in our setting. However, where approved contemporary alternatives are readily available and clinically appropriate, same-agent double dosing should not be presumed to be preferable. Because no direct comparative or economic analysis was performed, the relative efficacy, safety, durability, and cost-effectiveness of these strategies cannot be determined from the present study.

Worse baseline visual acuity was independently associated with a higher risk of subretinal fibrosis. This association is consistent with the possibility that poorer baseline vision reflects more advanced structural damage or a greater disease burden, although these mechanisms were not directly quantified in the present study [22,23,24]. Kaplan–Meier analyses showed that the cumulative probability of first complete OCT dryness increased mainly during the early to middle follow-up period, whereas macular atrophy events were uncommon and subretinal fibrosis occurred more frequently. These findings suggest that baseline visual status may have prognostic relevance for subsequent structural outcomes, but they should be interpreted as observational associations rather than evidence of a causal relationship.

Several limitations of this study should be acknowledged. First, the uncontrolled retrospective within-eye pre–post design lacked a contemporaneous standard-dose control group and was susceptible to regression to the mean, effects of ongoing treatment, natural fluctuations in disease activity, and time-dependent confounding. Accordingly, causal benefit or superiority over standard-dose treatment cannot be established from the present study.

Second, the cohort included multiple exudative macular diseases and three anti-VEGF agents. Although this heterogeneity reflects routine clinical practice, nAMD predominated and the non-nAMD and agent-specific subgroups were relatively small, limiting robust disease- and agent-specific conclusions. Third, because the exact cumulative number and dates of injections administered outside our institution were not consistently recoverable, prior anti-VEGF exposure could not be fully characterized and may have varied among eyes. In addition, persistent or recurrent exudative activity was the common eligibility feature, whereas insufficient visual response and inadequate durability were not separately coded, precluding reliable subgroup analyses based on these overlapping considerations.

Fourth, the timing of the final follow-up varied among eyes. Consequently, baseline-to-final comparisons and exploratory regression analyses may have been influenced by differences in follow-up duration, although the principal longitudinal mixed-effects analyses incorporated continuous time since dose escalation. Fifth, the paired injection-interval analysis was restricted to 73 eyes with calculable intervals during both treatment phases. Record availability was non-random, and the included and excluded eyes differed in several available demographic, clinical, and treatment characteristics (Supplementary Table S9). Residual selection bias therefore remains, its direction and magnitude cannot be determined with certainty, and the interval findings should not be generalized to the entire cohort.

Sixth, although IOP remained stable and no serious ocular adverse events were documented during follow-up, safety outcomes were identified retrospectively, and the sample size and follow-up duration were insufficient to exclude rare adverse events or support definitive safety conclusions. Finally, the single-center Chinese tertiary-care setting and the predominance of nAMD may limit the generalizability of the findings to other populations, institutions, diseases, and treatment settings.

Despite these limitations, the study has several strengths. First, it addresses a clinically relevant and relatively understudied scenario: same-agent dose escalation in eyes with a suboptimal response to standard-dose anti-VEGF therapy. Second, repeated within-eye measurements at the pre-switch baseline and four post-switch assessments enabled a consistent description of temporal changes while reducing between-eye variability in the descriptive comparisons, although this design does not establish causality. Third, the study evaluated complementary functional, anatomical, safety, durability, and prognostic outcomes, including BCVA, CST, IOP, OCT biomarkers, injection intervals, time-to-event outcomes, and exploratory agent-stratified analyses. Together, these features provide a broad real-world characterization of the observed outcomes and limitations of same-agent double-dose escalation in routine clinical practice.

## 5. Conclusions

In this uncontrolled real-world pre–post cohort, anatomical changes after same-agent double-dose anti-VEGF escalation were more consistent than functional changes. CST remained lower throughout post-switch follow-up, whereas the longitudinal BCVA change was small and of uncertain clinical relevance. No significant interval extension was observed in the 73-eye complete-case subgroup during the relatively short follow-up; however, heterogeneity in durability outcomes across previous intensified anti-VEGF studies means that a longer-term benefit cannot be excluded [11,13,16,21]. Same-agent double-dose escalation may therefore represent a context-dependent, off-label option when approved high-dose or dual-pathway alternatives are unavailable or clinically unsuitable, rather than a universally preferred intensification strategy [10,14,15]. Exploratory analyses suggested that baseline PED was associated with a less favorable BCVA change and that worse baseline visual acuity was associated with a higher risk of subretinal fibrosis, broadly consistent with previous evidence [20,22,23,24]. Prospective controlled studies with longer follow-up and protocolized interval extension are needed.

## Figures and Tables

**Table 1:** Baseline demographic and clinical characteristics.

Variable	Overall (n = 111 Eyes)
Demographic characteristics	
Age, years	71 (60.5, 78.0)
Patient sex, n (%)	
Male	69 (62.2)
Female	42 (37.8)
Disease characteristics	
Diagnosis, n (%)	
nAMD	87 (78.4)
DME	10 (9.0)
RVO-CME	8 (7.2)
Other CNV	6 (5.4)
Study eye, n (%)	
Right eye (OD)	63 (56.8)
Left eye (OS)	48 (43.2)
Pre-switch baseline status	
BCVA (logMAR)	0.7 (0.4, 1.0)
IOP, mmHg	14.0 (12.20, 15.60)
History of standard-dose anti-VEGF therapy before switching	
Treatment duration, months	8 (2, 16)
Initial same-agent double-dose regimen, n (%)	
Aflibercept, 2.0 → 4.0 mg	72 (64.9)
Conbercept, 0.5 → 1.0 mg	28 (25.2)
Ranibizumab, 0.5 → 1.0 mg	11 (9.9)
Post-switch follow-up duration, months	6.9 (5.7, 9.3)

Continuous variables are presented as median (Q1, Q3), and categorical variables as n (%). Unless otherwise stated, characteristics are summarized at the eye level; patients contributing both eyes are represented for each included eye. All included eyes had received at least three prior standard-dose anti-VEGF injections. Exact cumulative injection counts were not consistently recoverable for eyes previously treated outside our institution and were therefore not reported. nAMD, neovascular age-related macular degeneration; DME, diabetic macular edema; RVO-CME, retinal vein occlusion-related cystoid macular edema; CNV, choroidal neovascularization; OD, right eye; OS, left eye; logMAR, logarithm of the minimum angle of resolution; BCVA, best-corrected visual acuity; IOP, intraocular pressure; anti-VEGF, anti-vascular endothelial growth factor.

**Table 2:** Functional and anatomical outcomes before and after switching to double-dose anti-VEGF therapy.

Variable	Pre-Switch Baseline (n = 111)	First Post-Switch Follow-Up (n = 111)	Second Post-Switch Follow-Up (n = 111)	Third Post-Switch Follow-Up (n = 111)	Final Follow-Up (n = 111)
**Functional outcomes**					
Best-corrected visual acuity (logMAR)	0.70 (0.40, 1.00)	0.82 (0.40, 1.00) *p* = 0.776	0.82 (0.40, 1.22) *p* = 0.608	0.70 (0.40, 1.10) *p* = 0.685	0.70 (0.40, 1.00) *p* = 0.004
Change in BCVA from baseline	NA	0.00 (−0.10, 0.10)	0.00 (−0.11, 0.12)	0.00 (−0.19, 0.31)	0.00 (−0.25, 0.10)
**Anatomical outcomes**					
Central subfield thickness (CST, μm)	282 (221, 361)	262 (212.4, 340.5) *p* = 0.002	252 (216.85, 311.00) *p* = 0.004	238 (205.5, 294.0) *p* < 0.001	236 (207.0, 294.5) *p* < 0.001
Change in CST from baseline	NA	−9 (−62.5, 12.0)	−11 (−57, 19)	−27 (−80.5, 11.0)	−19 (−98.0, 3.5)
Intraretinal fluid (IRF) positive, n (%)	62 (55.9)	54 (48.6) *p* = 0.013	54 (48.6) *p* = 0.099	54 (48.6) *p* = 0.118	50 (45.0) *p* = 0.006
Subretinal fluid (SRF) positive, n (%)	49 (44.1)	38 (34.2) *p* = 0.046	38 (34.2) *p* = 0.054	40 (36.0) *p* = 0.188	37 (33.3) *p* = 0.059
Pigment epithelial detachment (PED) positive, n (%)	32 (28.8)	25 (22.5) *p* = 0.023	27 (24.3) *p* = 0.131	28 (25.2) *p* = 0.386	26 (23.4) *p* = 0.211

Continuous variables are presented as median (Q1, Q3), and categorical variables are presented as n (%). *p* values indicate comparisons between each post-switch follow-up visit and the pre-switch baseline. Continuous variables were compared using the Wilcoxon signed-rank test, and categorical variables were compared using the McNemar test. Negative BCVA changes indicate improvement in logMAR visual acuity, and negative CST changes indicate anatomical reduction. Visit-specific comparisons were considered secondary descriptive analyses.

## Data Availability

The datasets used and/or analyzed during the current study are available from the corresponding author on reasonable request.

## Supplementary Materials

The following supporting information can be downloaded at: https://www.mdpi.com/article/10.3390/jcm15155898/s1, Table S1: Mean values of functional and anatomical outcomes before and after switching to double-dose anti-VEGF therapy; Table S2: Comparison of injection intervals before and after switching to double-dose therapy; Table S3: Linear mixed-effects model for longitudinal BCVA (logMAR) after switching to double-dose anti-VEGF therapy; Table S4: Gamma mixed-effects model for longitudinal CST after switching to double-dose anti-VEGF therapy; Table S5: Cox regression analyses for time-to-event outcomes; Table S6: Multivariable linear regression analyses; Table S7: Descriptive achievement of complete OCT dryness by disease category; Table S8: Exploratory outcomes stratified by initial double-dose anti-VEGF agent; Table S9: Characteristics of eyes included in and excluded from the paired injection-interval analysis; Figure S1: Kaplan–Meier curve for MA-free survival after switching to double-dose anti-VEGF therapy.

## Abbreviations

anti-VEGF	Anti-vascular endothelial growth factor
BCVA	Best-corrected visual acuity
CI	Confidence interval
CNV	Choroidal neovascularization
CST	Central subfield thickness
DME	Diabetic macular edema
HF	Hyperreflective foci
HR	Hazard ratio
IOP	Intraocular pressure
IRF	Intraretinal fluid
IQR	Interquartile range
logMAR	Logarithm of the minimum angle of resolution
MA	Macular atrophy
nAMD	Neovascular age-related macular degeneration
OCT	Optical coherence tomography
PED	Pigment epithelial detachment
RVO-CME	Retinal vein occlusion-related cystoid macular edema
SD	Standard deviation
SRF	Subretinal fluid

## References

[1] Fleckenstein M., Schmitz-Valckenberg S., Chakravarthy U. (2024). Age-related macular degeneration: A review. JAMA.

[2] Browning D.J., Stewart M.W., Lee C. (2018). Diabetic macular edema: Evidence-based management. Indian J. Ophthalmol..

[3] Laouri M., Chen E., Looman M., Gallagher M. (2011). The burden of disease of retinal vein occlusion: Review of the literature. Eye.

[4] Pham B., Thomas S.M., Lillie E., Lee T., Hamid J., Richter T., Janoudi G., Agarwal A., Sharpe J.P., Scott A. (2019). Anti-vascular endothelial growth factor treatment for retinal conditions: A systematic review and meta-analysis. BMJ Open.

[5] Nichani P.A.H., Popovic M.M., Mihalache A., Pathak A., Muni R.H., Wong D.T.W., Kertes P.J. (2024). Efficacy and safety of intravitreal faricimab in neovascular age-related macular degeneration, diabetic macular edema, and retinal vein occlusion: A meta-analysis. Ophthalmologica.

[6] Broadhead G.K., Phan L., Keenan T.D.L., Chin J., Hong T., Chew E.Y., Chang A.A. (2026). How to judge failure: Defining treatment resistance to anti-VEGF therapy in exudative maculopathies—A systematic scoping review. Ophthalmol. Retin..

[7] Savant S.V., Kwan J.T., Barouch F., Chang J., Ramsey D.J., Marx J., Blaha G., Klein-Mascia K. (2024). Durability and efficacy of faricimab in treatment-resistant retinal edema utilizing real-world dosing regimens. J. Ophthalmol..

[8] Hutton-Smith L.A., Gaffney E.A., Byrne H.M., Maini P.K., Schwab D., Mazer N.A. (2016). A mechanistic model of the intravitreal pharmacokinetics of large molecules and the pharmacodynamic suppression of ocular vascular endothelial growth factor levels by ranibizumab in patients with neovascular age-related macular degeneration. Mol. Pharm..

[9] Zhu W., Hao Y., Yuan Z., Huang C., Liu J., Ma Y. (2023). Long-term outcomes of high-dose conbercept treatment for myopic choroidal neovascularization and idiopathic choroidal neovascularization. Ophthalmic Res..

[10] Wykoff C.C., Brown D.M., Reed K., Berliner A.J., Gerstenblith A.T., Breazna A., Abraham P., Fein J.G., Chu K.W., Clark W.L. (2023). Effect of high-dose intravitreal aflibercept, 8 mg, in patients with neovascular age-related macular degeneration: The phase 2 CANDELA randomized clinical trial. JAMA Ophthalmol..

[11] Broadhead G.K., Keenan T.D.L., Chew E.Y., Wiley H.E., Cukras C.A. (2022). Comparison of agents using higher dose anti-VEGF therapy for treatment-resistant neovascular age-related macular degeneration. Graefes Arch. Clin. Exp. Ophthalmol..

[12] Qian T., Zhou Y., Zhou H., Wu W., Yuan Y., Yu S., Xu X. (2025). Intravitreal double-dose conbercept injection for the treatment of neovascular age-related macular degeneration: A pilot real-life clinical practice study. Clin. Ophthalmol..

[13] Zhang M., Liu X., Gong Y., Qian T., Zhou H., Wang Y., Wu J., Sun X., Yu S. (2024). Double-dose investigation of aflibercept in neovascular age-related macular degeneration (DIANA): A real-world study. BMC Ophthalmol..

[14] Lanzetta P., Korobelnik J.F., Heier J.S., Leal S., Holz F.G., Clark W.L., Eichenbaum D., Iida T., Xiaodong S., Berliner A.J. (2024). Intravitreal aflibercept 8 mg in neovascular age-related macular degeneration (PULSAR): 48-week results from a randomised, double-masked, non-inferiority, phase 3 trial. Lancet.

[15] Heier J.S., Khanani A.M., Quezada Ruiz C., Basu K., Ferrone P.J., Brittain C., Figueroa M.S., Lin H., Holz F.G., Patel V. (2022). Efficacy, durability, and safety of intravitreal faricimab up to every 16 weeks for neovascular age-related macular degeneration (TENAYA and LUCERNE): Two randomised, double-masked, phase 3, non-inferiority trials. Lancet.

[16] Bala S., Barbosa G.C.S., Mohan N., Srivastava S.K., Kaiser P.K., Sastry A., Babiuch A.S., Sears J., Talcott K.E., Yuan A. (2025). Initial functional and anatomical outcomes of high-dose aflibercept 8 mg in exudative neovascular age-related macular degeneration. Ophthalmol. Retin..

[17] Simader C., Ritter M., Bolz M., Deák G.G., Mayr-Sponer U., Golbaz I., Kundi M., Schmidt-Erfurth U. (2014). Morphologic parameters relevant for visual outcome during anti-angiogenic therapy of neovascular age-related macular degeneration. Ophthalmology.

[18] Steinle N.C., McCullough A.J., Silva F.Q., Du W., Moini H., Singh R.P. (2025). Impact of duration of exposure to intraretinal fluid on visual outcomes in neovascular age-related macular degeneration. Ophthalmol. Retin..

[19] Chaudhary V., Matonti F., Zarranz-Ventura J., Stewart M.W. (2022). Impact of fluid compartments on functional outcomes for patients with neovascular age-related macular degeneration: A systematic literature review. Retina.

[20] Nanji K., Grad J., Hatamnejad A., McKechnie T., Phillips M., Cheung C.M.G., Patel P.J., Marco R.D., Borrelli E., Steel D.H. (2025). Baseline OCT biomarkers predicting visual outcomes in neovascular age-related macular degeneration: A meta-analysis. Ophthalmology.

[21] Funatsu M., Higashijima F., Ariyoshi N., Haraguchi A., Wasai Y., Mikuni M., Ohta M., Wakuta M., Hirano S., Yamauchi K. (2026). Switching to high-dose aflibercept (8 mg) with pro re nata reduces treatment burden in diabetic macular edema: A real-world pilot study. J. Clin. Med..

[22] Wu J., Zhang J. (2022). Neovascular remodeling and subretinal fibrosis as biomarkers for predicting incomplete response to anti-VEGF therapy in neovascular age-related macular degeneration. Front. Biosci. (Landmark Ed.).

[23] Cheong K.X., Cheung C.M.G., Teo K.Y.C. (2023). Review of fibrosis in neovascular age-related macular degeneration. Am. J. Ophthalmol..

[24] Liu S., Zheng M., Sun H., Pan C., Li D., Zhou X., Zheng Z. (2024). Analysis of factors affecting prognosis of the visual acuity and baseline risk factors for subretinal fibrosis in neovascular age-related macular degeneration patients. Front. Med..

[25] Bleidißel N., Weichenberger M., Maier M., Spielberg N., Feucht N. (2025). Improved functional and morphological outcomes with faricimab in nAMD eyes with poor response to prior intravitreal anti-VEGF therapy. Int. Ophthalmol..

[26] Leung E.H., Oh D.J., Alderson S.E., Bracy J., McLeod M., Perez L.I., Bottini A., Yee D.C., Mukkamala K. (2023). Initial real-world experience with faricimab in treatment-resistant neovascular age-related macular degeneration. Clin. Ophthalmol..

[27] Heinke A., Warter A., Nagel I.D., Agnihotri A., Mehta N.N., Galang C.M.B., Deussen D.N., Bartsch D.-U.G., Cheng L., Ferreyra H.A. (2025). Faricimab for treatment-resistant choroidal neovascularization in neovascular age-related macular degeneration: Seven-month results using artificial intelligence and OCTA. Int. J. Retin. Vitr..

[28] Nagel I.D., Tran M.D., Zhang H., Cheng L., Heinke A., Mehta N.N., Bartsch D.U., Kalaw F.G.P., Morsy M., Mueller A.J. (2025). Faricimab at 6 and 12 mg reduces pigment epithelium detachment in treatment-resistant macular neovascularization: An OCT and AI analysis. Sci. Rep..

[29] Bayer Eylea™ 8 mg Approved in China for Wet Age-Related Macular Degeneration. https://www.bayer.com/media/en-us/eylea-8-mg-approved-in-china-for-wet-age-related-macular-degeneration/.

