# Supplementary material for: Clinical Outcomes After Same-Agent Double-Dose Anti-VEGF Escalation in Eyes with a Suboptimal Response to Standard-Dose Treatment for Exudative Macular Diseases: A Real-World Pre–Post Study
Source: J Clin Med. 2026 Jul 28;15(15):5898. doi: 10.3390/jcm15155898 (PMC13466651; doi:10.3390/jcm15155898)
Supplement: Supplementary file 1 [file jcm-15-05898-s001.zip › jcm-4396091-supplementary.pdf]

Supplementary Table S1. Mean values of functional and anatomical outcomes before and after switching to double-dose anti-VEGF therapy

Variable	**Pre-switch baseline (n = 111)**	**First post-switch follow-up (n = 111)**	**Second post-switch follow-up (n = 111)**	**Third post-switch follow-up (n = 111)**	**Final follow-up (n = 111)**
**Functional outcomes**					
Best-corrected visual acuity (logMAR)	0.78±0.45	0.79±0.46	0.81±0.50	0.77±0.45	0.70±0.38
Change in BCVA from baseline	-	0.01±0.23	0.03±0.34	-0.01±0.32	-0.07±0.30
Anatomical outcomes					
Central subfield thickness (CST, μm)	327.06±182.23	298.32±150.51	295.27±159.36	271.73± 130.05	271.94±130.51
Change in CST from baseline	-	-28.74±117.76	-31.79±149.69	-55.33±137.44	-55.12±174.02
Time since previous injection at each follow-up visit, days	-	60.04±30.49	58.24±29.94	59.1±28.87	59.36±33.45
Time since baseline injection at each follow-up visit, days	-	60.04±30.49	118.24±54.28	177.38±71.96	236.77±89.53

Continuous variables are presented as mean ± standard deviation.

Supplementary Table S2. Comparison of injection intervals before and after switching to double-dose therapy

	Standard-dose phase (n = 73)	Double-dose phase (n = 73)	P value
Injection interval, days, median (IQR)	55 (38, 85)	52 (43, 69)	0.289

Data are presented as median (Q1, Q3). Injection intervals were calculated at the eye level. For the standard-dose phase, the mean interval was calculated from the period between the first standard-dose injection documented at our institution and the first double-dose injection, divided by the number of standard-dose injections administered at our institution before switching. For the double-dose phase, the mean interval was calculated across four consecutive intervals defined by the switching injection and the first, second, third, and final post-switch injection visits. The paired analysis included 73 eyes with complete and accurately dated injection data in both phases. The P value was calculated using the paired Wilcoxon signed-rank test. IQR, interquartile range.

Supplementary Table S3. Linear mixed-effects model for longitudinal BCVA (logMAR) after switching to double-dose anti-VEGF therapy

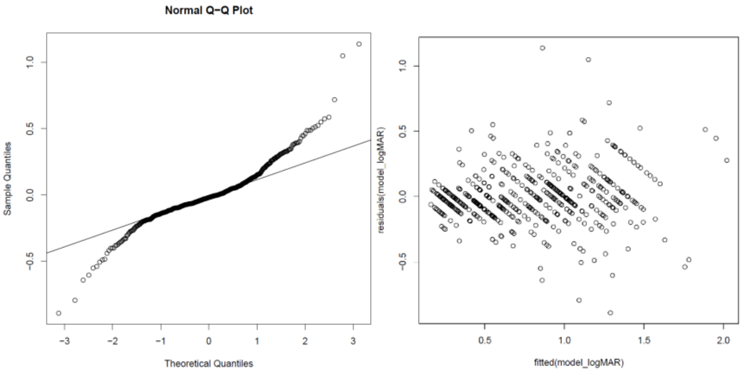

Variable	Beta	SE	P value
Time (month)	-0.007	0.003	0.028

Time since dose escalation, measured in months, was included as a continuous fixed effect. Eye-specific random intercepts and random slopes for time were included to account for repeated measurements within eyes. The model was fitted using the lmer() function in the lme4 package, with no additional fixed effects.

Supplementary Table S4. Gamma mixed-effects model for longitudinal CST after switching to double-dose anti-VEGF therapy

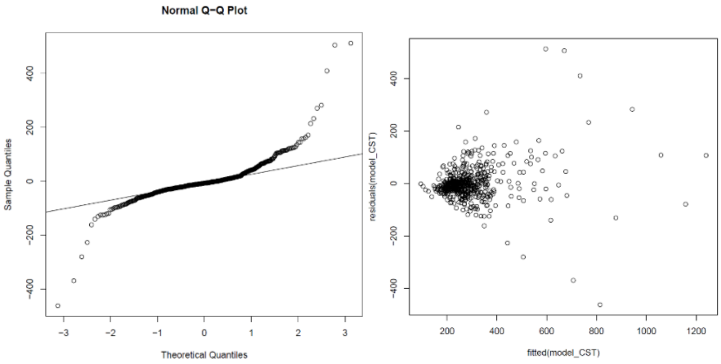

Variable	Beta	exp(Beta)	SE	P value
Time (month)	-0.021297	0.979	0.005112	< 0.001

Time since dose escalation, measured in months, was included as a continuous fixed effect. Eye-specific random intercepts and random slopes for time were included to account for repeated measurements within eyes. The model was fitted using the glmmTMB() function in the glmmTMB package, with a gamma distribution and log link and no additional fixed effects.

Supplementary Table S5. Cox regression analyses for time-to-event outcomes

Panel A. Factors associated with time to first complete OCT dryness

n = 110, number of events = 46

Variable	HR	95% CI	P value
Baseline age	1.006	0.978-1.035	0.67
Male (vs Female)	0.707	0.386-1.295	0.261
Baseline logMAR	1.066	0.566-2.007	0.843
Baseline CST	0.999	0.997-1.001	0.32
Baseline IOP	0.888	0.776-1.016	0.083
Baseline MA	0.744	0.175-3.163	0.689
Baseline subretinal fibrosis	0.641	0.289-1.423	0.274

For Panel A, complete OCT dryness was defined as the absence of intraretinal fluid, subretinal fluid, and pigment epithelial detachment on OCT.

Panel B. Factors associated with time to first observed macular atrophy

n = 110, number of events = 10

Variable	HR	95% CI	P value
Baseline age	1.007	0.942-1.077	0.832
Male (vs Female)	0.661	0.165-2.649	0.558
Baseline logMAR	0.326	0.049-2.181	0.248
Baseline CST	0.993	0.983-1.003	0.179
Baseline IOP	1.083	0.891-1.316	0.422
Baseline SRF	NE	NE	NE
Baseline PED	1.344	0.254-7.124	0.728
Baseline subretinal fibrosis	4.514	0.878-23.196	0.071

NE, not estimable because of sparse events or model instability.

Panel C. Factors associated with time to first observed subretinal fibrosis

n = 110, number of events = 36

Variable	HR	95% CI	P value
Baseline age	1.019	0.983-1.055	0.306
Male (vs Female)	0.734	0.369-1.462	0.379
Baseline logMAR	2.854	1.493-5.453	0.002
Baseline CST	0.997	0.994-1.000	0.086
Baseline IOP	1.014	0.883-1.164	0.847
Baseline SRF	0.493	0.225-1.081	0.077
Baseline PED	0.782	0.322-1.902	0.588
Baseline MA	1.456	0.415-5.106	0.557

The Cox regression analyses were based on 110 eyes because one eye had incomplete data for the time-to-event models.

Supplementary Table S6. Multivariable linear regression analyses

Panel A. Change in BCVA from baseline to final follow-up

Variable	Beta	SE	95% CI	P value
Baseline age	-0.002	0.003	(-0.007, 0.003)	0.410
Male (vs Female)	0.003	0.058	(-0.111, 0.116)	0.963
Baseline CST	-0.00015	0.00017	(-0.00048, 0.00017)	0.360
Baseline IOP	0.004	0.012	(-0.02, 0.027)	0.771
Baseline SRF	-0.070	0.061	(-0.19, 0.05)	0.258
Baseline PED	0.198	0.064	(0.073, 0.323)	0.002
Baseline MA	0.176	0.139	(-0.097, 0.449)	0.209
Baseline subretinal fibrosis	0.002	0.074	(-0.142, 0.147)	0.974

Panel B. Change in CST from baseline to final follow-up

Variable	Beta	SE	95% CI	P value
Baseline age	1.281	1.521	(-1.7, 4.263)	0.402
Male (vs Female)	-49.468	34.202	(-116.504, 17.569)	0.151
Baseline logMAR	-40.453	37.793	(-114.527, 33.622)	0.287
Baseline IOP	-4.839	7.131	(-18.815, 9.138)	0.499
Baseline SRF	-49.822	35.740	(-119.872, 20.228)	0.166
Baseline PED	-28.544	37.874	(-102.776, 45.688)	0.453
Baseline MA	-12.828	82.279	(-174.094, 148.439)	0.876
Baseline subretinal fibrosis	18.319	44.162	(-68.239, 104.877)	0.679

Supplementary Figure S1

Supplementary Figure S1. Kaplan–Meier curve for MA-free survival after switching to double-dose anti-VEGF therapy.

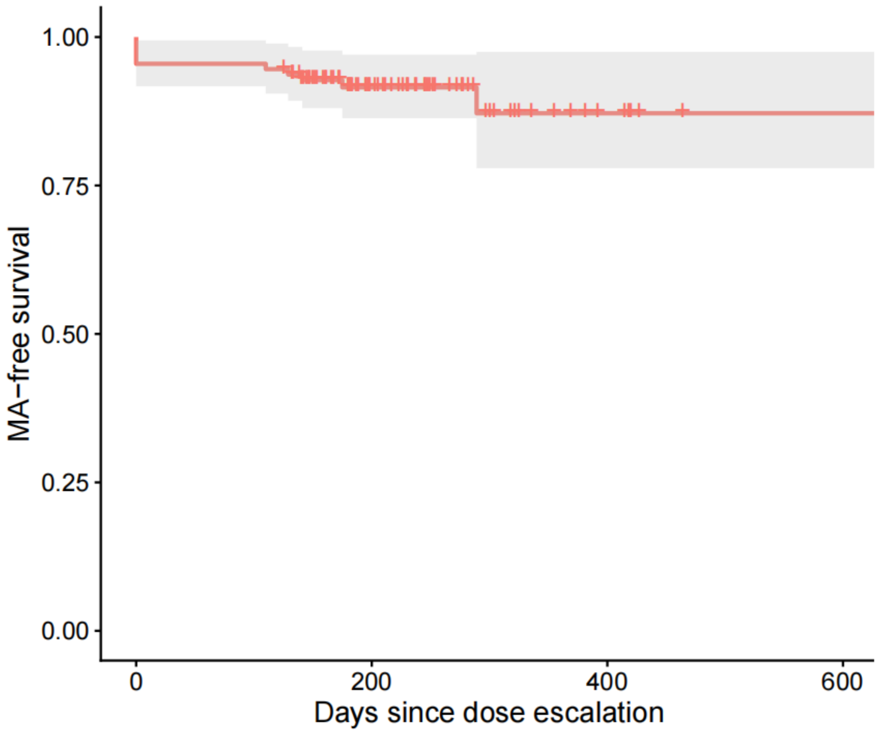

Supplementary Table S7. Descriptive achievement of complete OCT dryness by disease category

Disease category	Eyes with at least one of IRF, SRF, or PED at the pre-switch baseline, n	Complete OCT dryness during follow-up, n (%)
nAMD	73	25 (34.2)
DME	8	2 (25.0)
RVO-CME	8	4 (50.0)
Other CNV	6	1 (16.7)
Overall	95	32 (33.7)

Complete OCT dryness was defined as the absence of IRF, SRF, and PED at any post-switch OCT assessment among eyes with at least one of these features at the pre-switch baseline. No formal between-disease comparisons were performed because of the small subgroup sizes.

Supplementary Table S8. Exploratory outcomes stratified by initial double-dose anti-VEGF agent

Outcome	Aflibercept, n = 72	Conbercept, n = 28	Ranibizumab, n = 11	P value
Baseline BCVA, logMAR	0.70 (0.40, 1.00)	0.92 (0.58, 1.06)	0.40 (0.40, 0.85)	—
Final BCVA, logMAR	0.60 (0.40, 0.92)	0.87 (0.40, 1.00)	0.52 (0.40, 0.76)	—
BCVA change	0.00 (-0.22, 0.10)	-0.12 (-0.30, 0.00)	0.00 (-0.24, 0.12)	0.218
Baseline CST, μm	264.0 (214.8, 350.3)	310.0 (253.5, 363.3)	293.0 (256.5, 453.5)	—
Final CST, μm	237.5 (211.3, 297.5)	237.5 (205.5, 295.5)	235.0 (211.0, 262.0)	—
CST change, μm	-14.9 (-67.0, 10.0)	-25.5 (-139.2, -5.0)	-72.0 (-142.2, -23.5)	0.134

Values are presented as median (interquartile range). Changes were calculated as final follow-up minus baseline; negative BCVA changes indicate improvement in logMAR visual acuity, and negative CST changes indicate anatomical reduction. Between-agent comparisons were performed using the Kruskal–Wallis test and should be considered exploratory.

Supplementary Table S9. Characteristics of eyes included in and excluded from the paired injection-interval analysis

Characteristic	Included (n = 73 eyes)	Excluded (n = 38 eyes)	Effect size
Demographic characteristics			
Age at dose escalation, years	72.3 (64.0, 79.3)	70.0 (59.9, 76.6)	0.171*
Male sex, n (%)	50 (68.5)	19 (50.0)	0.383
Right eye, n (%)	36 (49.3)	27 (71.1)	0.455
Disease characteristics			
nAMD, n (%)	58 (79.5)	29 (76.3)	0.076
DME, n (%)	7 (9.6)	3 (7.9)	0.060
RVO-CME, n (%)	5 (6.8)	3 (7.9)	0.040
Other CNV, n (%)	3 (4.1)	3 (7.9)	0.160
Pre-switch clinical and OCT characteristics			
Pre-switch BCVA, logMAR	0.8 (0.4, 1.0)	0.6 (0.3, 1.0)	0.152*
Pre-switch IOP, mmHg	13.9 (12.1, 15.4)	14.4 (12.5, 16.1)	0.132*
Pre-switch CST, μm	263.0 (221.0, 361.0)	313.0 (227.5, 360.5)	0.137*
IRF present, n (%)	40 (54.8)	22 (57.9)	0.063
SRF present, n (%)	30 (41.1)	19 (50.0)	0.180
PED present, n (%)	22 (30.1)	10 (26.3)	0.085
MA present, n (%)	3 (4.1)	2 (5.3)	0.055

Subretinal fibrosis present, n (%)	15 (20.5)	7 (18.4)	0.054
Treatment characteristics			
Documented standard-dose treatment duration, months	5.9 (2.3, 11.2)	12.2 (8.0, 21.5)	0.398*
Initial aflibercept escalation, n (%)	41 (56.2)	31 (81.6)	0.571
Initial conbercept escalation, n (%)	23 (31.5)	5 (13.2)	0.452
Initial ranibizumab escalation, n (%)	9 (12.3)	2 (5.3)	0.251
Post-switch follow-up duration, months	6.7 (5.7, 9.1)	6.9 (5.6, 9.3)	0.072*

Continuous variables are presented as median (Q1, Q3), and categorical variables as n (%). Effect sizes marked with an asterisk (*) represent absolute Cliff's delta values for continuous variables; unmarked effect sizes represent absolute standardized mean differences (SMDs) for categorical variables. Cliff's delta was interpreted as negligible (<0.147), small (0.147 to <0.330), medium (0.330 to <0.474), or large (≥0.474), whereas an absolute SMD of ≥0.20 was considered potentially meaningful. The asterisk denotes the type of effect-size measure and does not indicate statistical significance. Documented standard-dose treatment duration was available for 73 included eyes and 37 excluded eyes.
